# On the Treatment of Diphtheria with Hyposulphite of Soda

**Published:** 1866-09

**Authors:** J. Clarkson Maynard

**Affiliations:** Wisbeach


### On the Treatment of Diphtheria with Hyposulphite of Soda.

BY J. CLARKSON MAYNARD, ESQ., WISBEACH.

On first visiting a case, if not very far advanced, and in which only a few spots are visible, the throat is dressed twice a-day out with a strong solution of the hyposulphite of soda, viz: ʒiij. of the hyposulphite, glycerine ʒij., with ʒvj. of water. This generally removes the incipient exudation in forty-eight hours, some times in less. But if the case is an advanced one and the parasite plant is making rapid strides, we wash the throat well out with warm water, by means of one of Maw's flexible syringes. This is alike agreeable and most beneficial to the patient. The affected parts are then dressed with the strong solution, and a gargling of ʒss. of the hyposulphite to half a pint of water, with ℥ss. of glycerine, is given, to be used every hour.

The effect of the solution upon the exudation is most marked. It appears to solidify and dry up the false membrane, and when the syringe is again used, which is to be frequently done, the force of water will, if not completely, nearly entirely wash it away. The exudation in this way seldom or ever reforms, and the patient makes comparatively a rapid recovery. In cases of a graver character, and where there is a larger collection than usual of inspissated mucus, we clear out the posterior nares by means of a powerful curved leaden syringe which is introduced into the nostril. In the putrid stage, and when the unpleasant odor from the throat is very offensive, a small quantity of Condy's disinfecting fluid added to the water with which we syringe the part has proved of great advantage. I may add that from half a gallon to a gallon of warm water ought, certainly in bad cases, to be thrown into the throat three or four times a day. The ext. belladonnæ applied externally has proved very useful where there has been much swelling.

In cases of very young children where it is difficult to dress and get at the throat, we give the hyposulphate internally, from gr. j. to gr. iij. every four hours, and allow them to swallow the gargle, which, by the way, they very frequently do without permission. Dr. Tubbs informs me he is now giving to adults gr. viij. every four hours. Port wine, beef-tea, brandy, and bark are, of course, given in suitable quantities, and in cases where there was much prostration we have occasionally thrown up, with very satisfactory results, an enema of port wine, beef-tea and isingglass.—*Medical Times and Gazette, Dec.* 30, 1865—*Braithwaite's Retrospect.*

